# Supplementary material for: The slow self-arresting nature of low-frequency earthquakes
Source: Nat Commun. 2021 Sep 15;12:5464. doi: 10.1038/s41467-021-25823-w (PMC8443596; doi:10.1038/s41467-021-25823-w)
Supplement: Supplementary file 1 — Supplementary Information for: The slow self-arresting nature of low-frequency earthquakes [file 41467_2021_25823_MOESM1_ESM.pdf]

Supplementary Information for

The slow self-arresting nature of low-frequency earthquakes

Xueting Wei[1,2], Jiankuan Xu[2,3], Yuxiang Liu[1,2], Xiaofei Chen[2, 4]*

Correspondence to: Xiaofei Chen (email: chenxf@sustech.edu.cn)

[1]School of Earth and Space Sciences, University of Science and Technology of China, Hefei, 230026, China.

[2]Department of Earth and Space Sciences, Southern University of Science and Technology, Shenzhen, 518055, China.

[3]Academy for Advanced Interdisciplinary Studies, Southern University of Science and Technology, Shenzhen, 518055, China.

[4]Shenzhen Key Laboratory of Deep Offshore Oil and Gas Exploration Technology, SUSTech, Shenzhen, 518055, China.

Supplementary Discussion

Effect of the nucleation method

An SSAR is a kind of rupture that arrests by itself before it breaks outside the nucleation zone. Here, we provide another nucleation method to study the inference on SSAR source parameters in this case.

Different from the nucleation mode of the whole nucleation zone breaking at the same time in the main text, we set the following initial stress so that the rupture starts from the centre of the nucleation zone and then grows to the whole nucleation zone with a rupture speed.

Referring to the nucleation method in Uenishi et al.'s work[1], we set the initial stress distribution in the nucleation zone as:

$$\begin{cases} T_0 = 0.9T_\mathrm{u} + 0.1T_\mathrm{u} \cdot t / (r / V_\mathrm{rup}) & T_0 \le T_\mathrm{u} \\ T_0 = T_\mathrm{u} + \delta T & T_0 > T_\mathrm{u} \end{cases} \quad (1),$$

where T_u is the breakdown stress drop defined in the slip-weakening law, δT is a small stress disturbance ($\delta T = 0.001T_\mathrm{u}$ in our simulations), t is the calculation time, r is the distance from the centre point of the nucleation zone to the calculation point and V_rup is the rupture velocity.

The initial stress distribution outside the nucleation zone is:

$$T_0 = T_\mathrm{e} \quad (2).$$

We set the nucleation patch radius as 100 m, P wave velocity as 6.000 km/s, S wave velocity as 3.464 km/s, density as 2.67 g/cm3, dynamic shear stress drop T_e as 1 MPa, and space step of 6 m for calculation. We simulate SSARs with a rupture patch diameter of 240 m and slip-weakening parameters $\hat{T}_\mathrm{e} = 0.9$, $\hat{D}_\mathrm{c} = 0.88$ and 0.92 ($D_\mathrm{c} = 3.66$ mm and 3.83 mm). Using the above frictional parameters, we conduct two sets of simulations with rupture velocities of 700 m/s and 3110 m/s (90% S wave velocity). The slip rate evolution is presented in Supplementary Fig. S12, and the source time function and frequency spectrum are shown in Supplementary Fig. S13.

For simulated SSARs with the same Dc value, the propagations of the slip rate are similar. The main differences lie in the time period before the crack covers the entire nucleation zone. For SSARs with rupture velocities of 700 m/s and 3110 m/s, the slip rate peaks are caused by local stress concentrations when the rupture front reaches each point in the nucleation zone. For SSARs with different Dc values, the propagation processes of ruptures are different. We also calculated the source parameters of these 6 earthquakes (Supplementary Table S3). For earthquakes with the same slip-weakening parameters, different rupture velocities mainly affect their source duration and average slip rate but have little effect on the stress drop and moment magnitude. Because the points in the nucleation area do not break at the same time, the source duration is

naturally longer than that of the case of simultaneous rupture in the nucleation zone. The slower the rupture velocity is, the longer the source duration is. Besides, the existence of rupture velocity will affect the spectrum characteristics of SSAR (Supplementary Fig. S13). The spectrum of SSAR with rupture velocity ∞ decays by f^{-1}, while the spectrums of SSAR with rupture 700 m/s and 3110 m/s decay by f^{-2}.

Compared with the slip rate of SSARs with rupture velocity ∞, the slip rate of SSARs with a rupture velocity of 700 m/s is reduced by 26% ($\hat{D}_c = 0.88$) and 35% ($\hat{D}_c = 0.92$). In contrast, the rupture velocity has a limited effect on the moment magnitude and stress drop (<5%). Regardless of the rupture velocity, the source parameters of these SSARs are still consistent with those of LFEs calculated from observations. Based on the numerical results above, we infer that setting different initial triggering methods has a limited effect on the formation and propagation of SSARs.

Fig. S1.

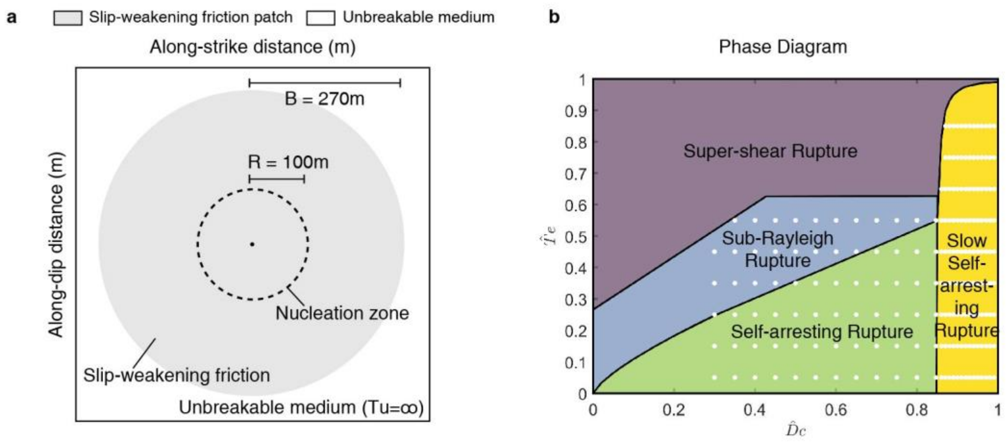

Numerical model of the Parkfield LFEs. a, Schematic diagram of the model geometry and frictional properties. The circular slip-weakening patch (grey) is embedded in an unbreakable fault domain. B is the asperity size of the slip-weakening patch, and R (the dotted circle) is the critical nucleation size of the slip-weakening patch to generate slip. **b**, Simulated slip-weakening parameters displayed in the phase diagram. White dots denote the locations of simulated $\left(\hat{T}_e, \hat{D}_c\right)$ pairs.

Fig. S2.

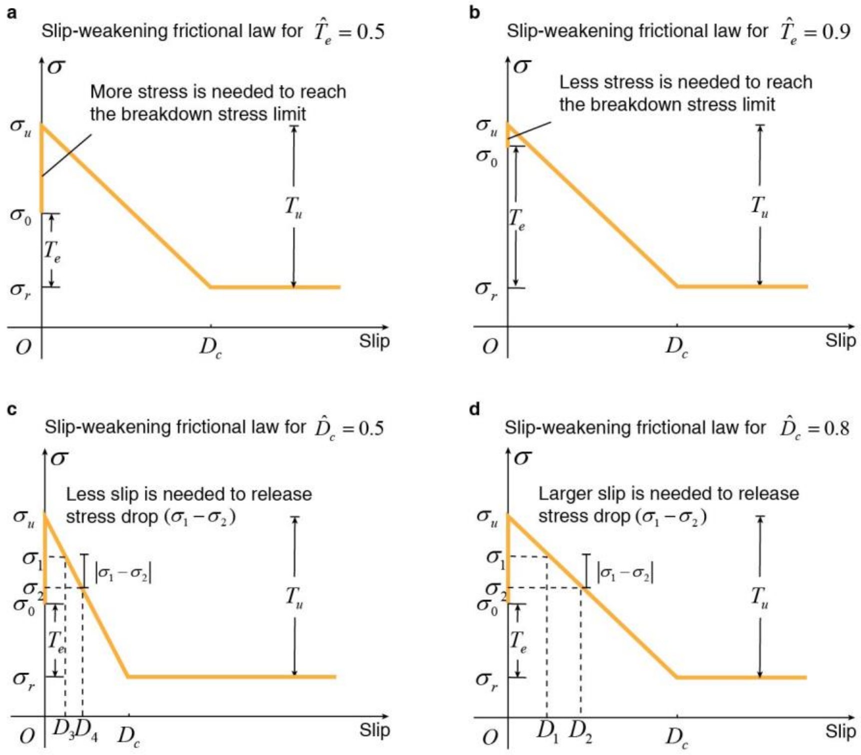

Effect of parameters $\hat{T}_\mathrm{e}$ and $\hat{D}_\mathrm{c}$ on the slip-weakening frictional law. With the same D_c value, rupture is more easily triggered under media with a larger $\hat{T}_\mathrm{e}$ value. With the same T_e value, a larger $\hat{D}_\mathrm{c}$ causes the stress release process in each seismic event to be less efficient. **a**, Slip-weakening law with $\hat{T}_\mathrm{e}$=0.5 and $\hat{D}_\mathrm{c}$=0.3. **b**, Slip-weakening law with $\hat{T}_\mathrm{e}$=0.9 and $\hat{D}_\mathrm{c}$=0.3. **c**, Slip-weakening law with $\hat{T}_\mathrm{e}$=0.5 and $\hat{D}_\mathrm{c}$=0.5. **d**, Slip-weakening law with $\hat{T}_\mathrm{e}$=0.5 and $\hat{D}_\mathrm{c}$=0.8.

Fig. S3.

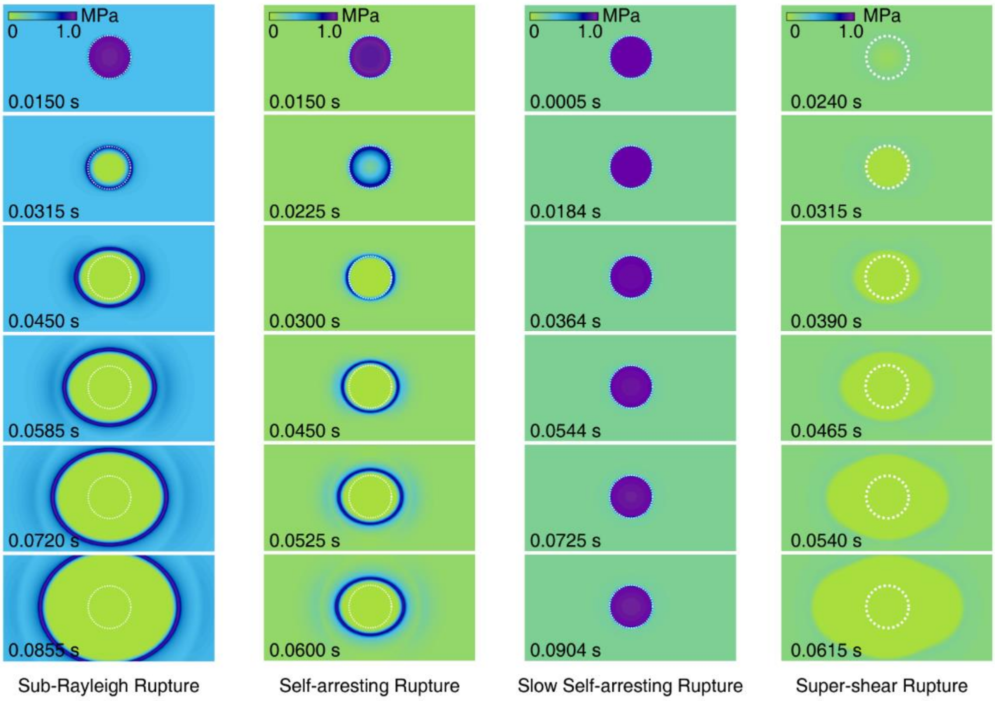

Stress evolution of four different rupture modes. The initial shear stress drop for all four types of ruptures is 1 MPa ($T_e = 1\text{MPa}$). The ruptures trigger at t=0 s within the area of the nucleation zone and propagate according to different friction properties. The frictional parameters used in the simulation are the same as in Fig. 1b. For the sub-Rayleigh rupture, $\hat{D}_c$ is 0.40, and $\hat{T}_e$ is 0.40; for the self-arresting rupture, $\hat{D}_c$ is 0.35, and $\hat{T}_e$ is 0.10; for the slow self-arresting rupture, $\hat{D}_c$ is 0.88, and $\hat{T}_e$ is 0.2; for the super-shear rupture, $\hat{D}_c$ is 0.40, and $\hat{T}_e$ is 0.80.

Fig. S4.

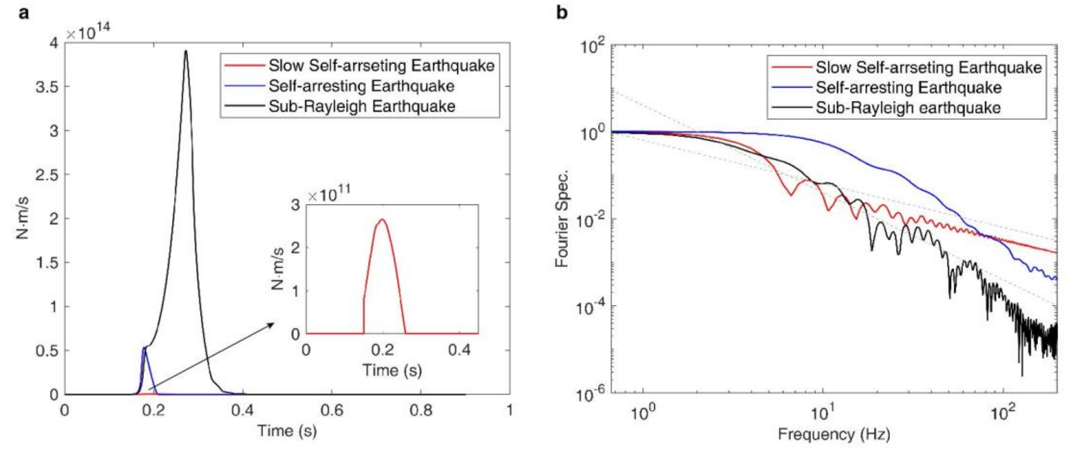

Source time function for the slow self-arresting earthquake and the corresponding Fourier spectra. a, Source time functions for slow self-arresting earthquakes, self-arresting earthquakes and sub-Rayleigh earthquakes. The simulation parameters are the same as in Figs. 1b and S3. **b**, Fourier spectra for slow self-arresting, self-arresting and sub-Rayleigh earthquakes. For a slow self-arresting earthquake, the spectra are well explained with a slope of f^{-1} rather than f^{-2} (dashed lines).

Fig. S5.

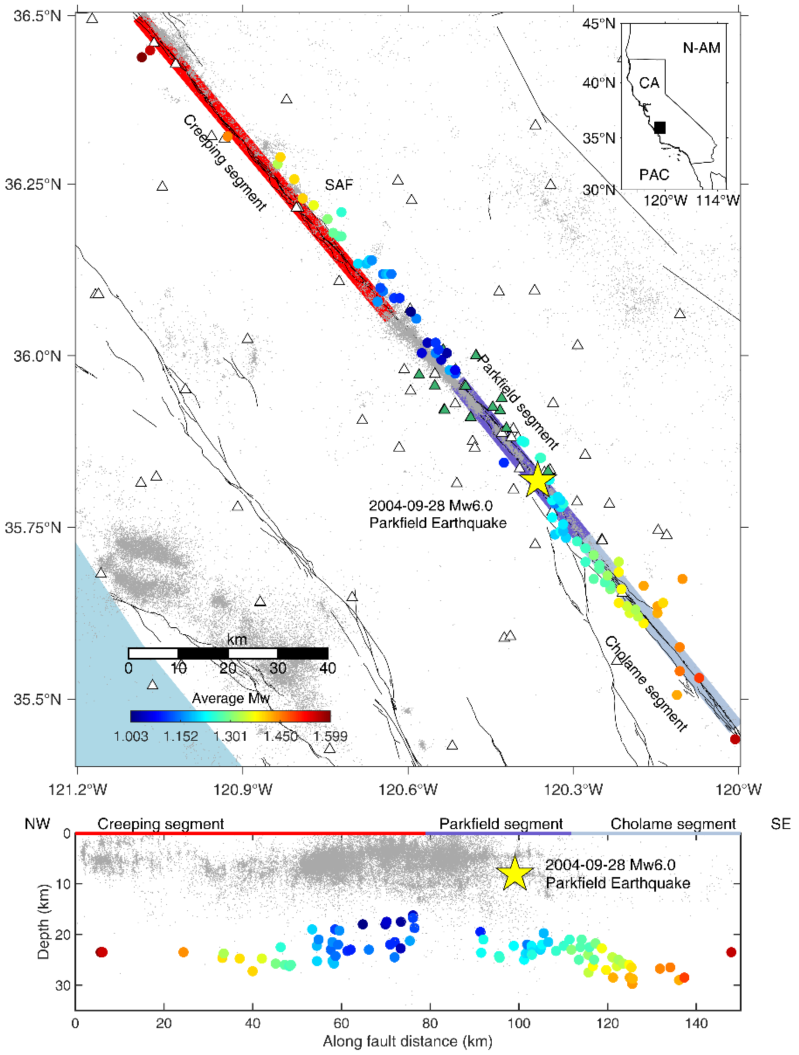

Map and LFE family locations and magnitudes. a, Locations of LFE (tremor) locations (coloured dots) and seismic stations (triangles). Filled triangles indicate borehole stations used for source parameter estimation. Grey dots show relocated micro seismicity. The yellow star indicates the hypocentre of the 2004 M 6 earthquake. Black lines show the faults. **b**, Along-fault section. The symbols are the same as in the map view.

Fig. S6.

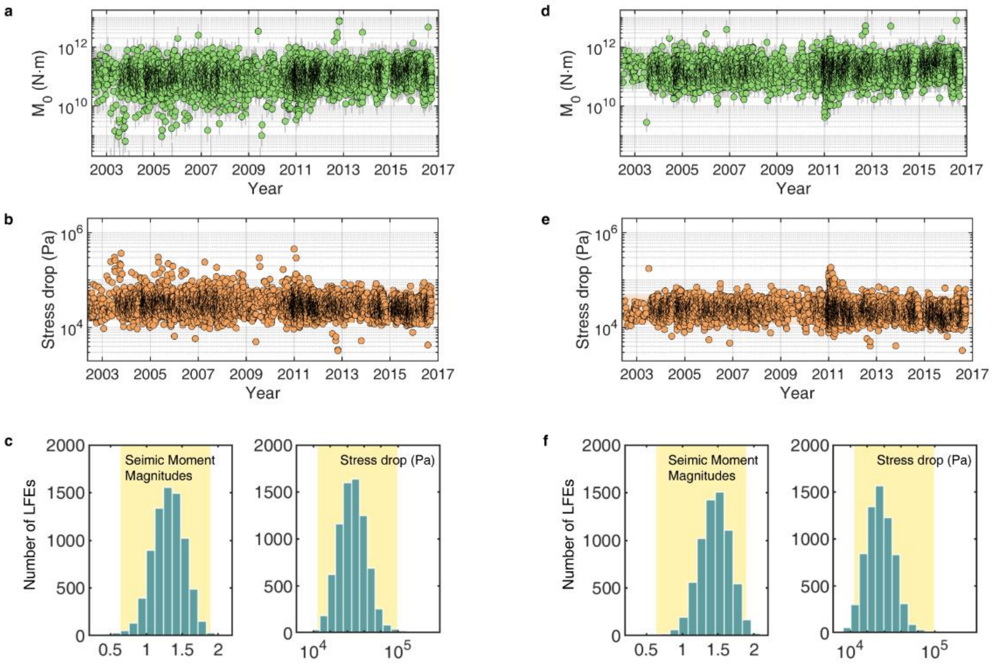

Seismic moments and stress drops of LFEs in Parkfield from 2002 to 2016 obtained with 2-8 Hz and 2-50 Hz filtered data. a, Seismic moments of LFEs obtained with 2-8 Hz filtered data (green dots). **b**, Stress drops of LFEs obtained with 2-8 Hz filtered data (orange dots). **c**, Histograms of seismic moment magnitudes and stress drops of LFEs obtained with 2-8 Hz filtered data. The yellow strips show the source parameter range we used in comparison with the simulated results. **d**, Seismic moment of LFEs obtained with 2-50 Hz filtered data. **e**, Stress drops of LFEs obtained with 2-50 Hz filtered data. **f**, Histograms of seismic moment magnitudes and stress drops of LFEs obtained with 2-50 Hz filtered data.

Fig. S7.

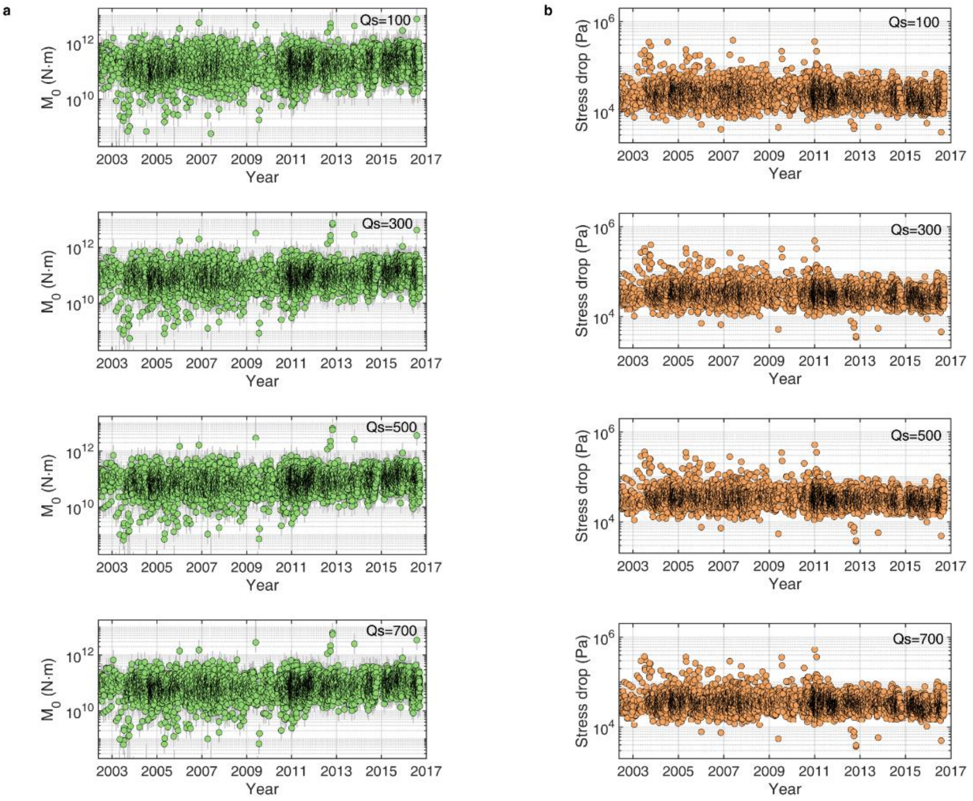

Seismic moments and stress drops of LFEs in Parkfield from 2002 to 2016 estimated with different Qs. a, The column from top to bottom shows the seismic moments of LFEs (green dots) obtained with Qs of 100, 300, 500 and 700. **b**, The column from top to bottom shows the stress drops of LFEs (orange dots) obtained with Qs of 100, 300, 500 and 700.

Fig. S8.

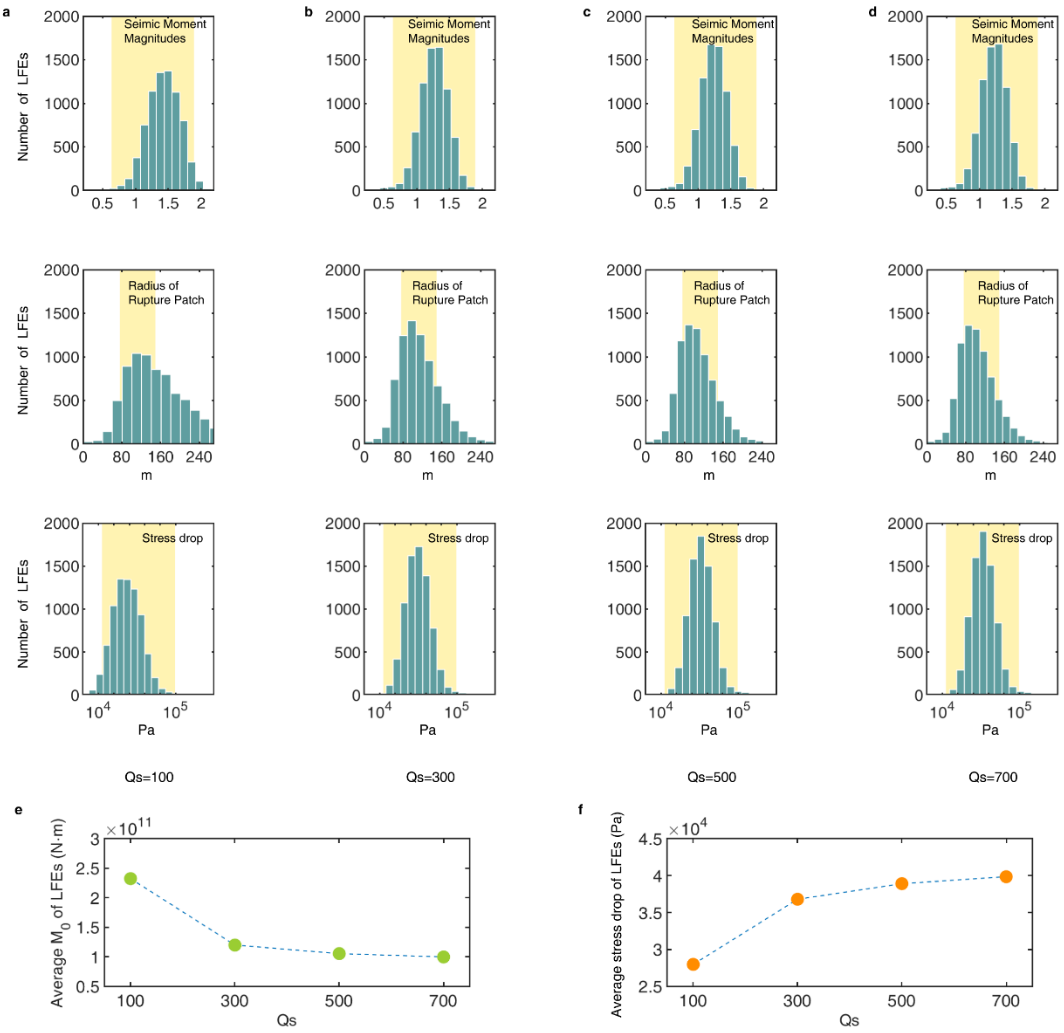

Comparison of moment magnitudes, rupture patch radii, and stress drops of LFEs obtained with different Qs. a, Histograms of LFE moment magnitudes, rupture patch radii and stress drops using Qs of 100 from top to bottom. The yellow strips show the source parameter range we used in comparison with the simulated results. **b**, Qs of 300. **c**, Qs of 500. **d**, Qs of 700. **e**, Comparison of the average seismic moments of LFEs in Parkfield (green dots) measured by using Qs of 100, 300, 500, 700. **f**, Comparison of the average stress drops of LFEs in Parkfield (orange dots) measured by using Qs of 100, 300, 500, 700.

Fig. S9.

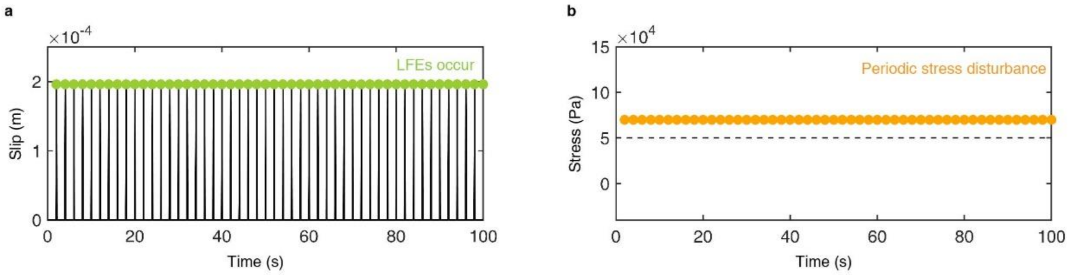

Recurrence of LFEs/SSARs under periodic stress disturbance. a, Slip evolution of the simulation LFE patch under the periodic shear stress disturbance. The green dot indicates the occurrence of each LFE. **b**, Introduced periodic stress disturbance.

Fig. S10.

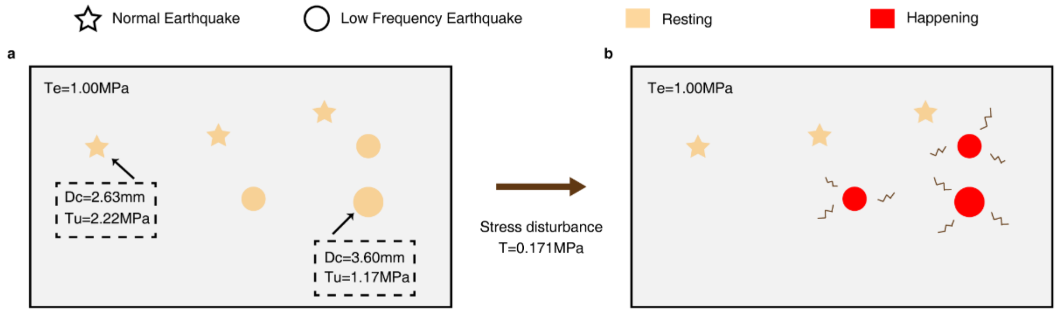

Modelled mechanism behind the LFEs. a, First, before any stress interference is introduced, the whole fault remains still. **b**, Stress disturbances reach the strength limit of SSAR nucleation patches. Therefore, LFEs occur, while other patches (nucleation patches of normal earthquakes) are far from reaching the stress limit.

Fig. S11.

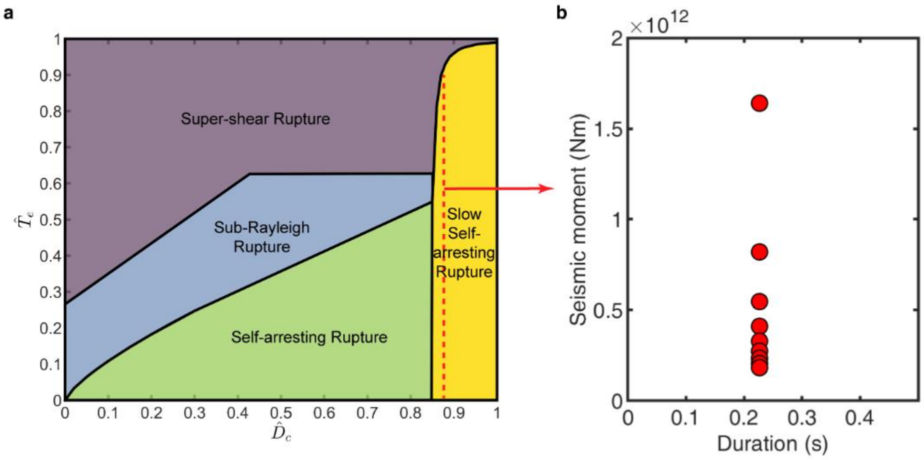

Moment-duration scaling relationship of SSARs with the same Dc. a, Phase diagram of rupture dynamics. The red dashed lines denote that the points in Fig. b have the same $\hat{D}_c$ value of 0.88, and their $\hat{T}_e$ values range from 0.1 to 0.9. **b**, The red dots represent LFEs with $\hat{T}_e$ ranging from 0.1 to 0.9. The diameter of the rupture zone of all simulated LFEs is 240 m, and $\hat{D}_c$ is 0.88.

Fig. S12.

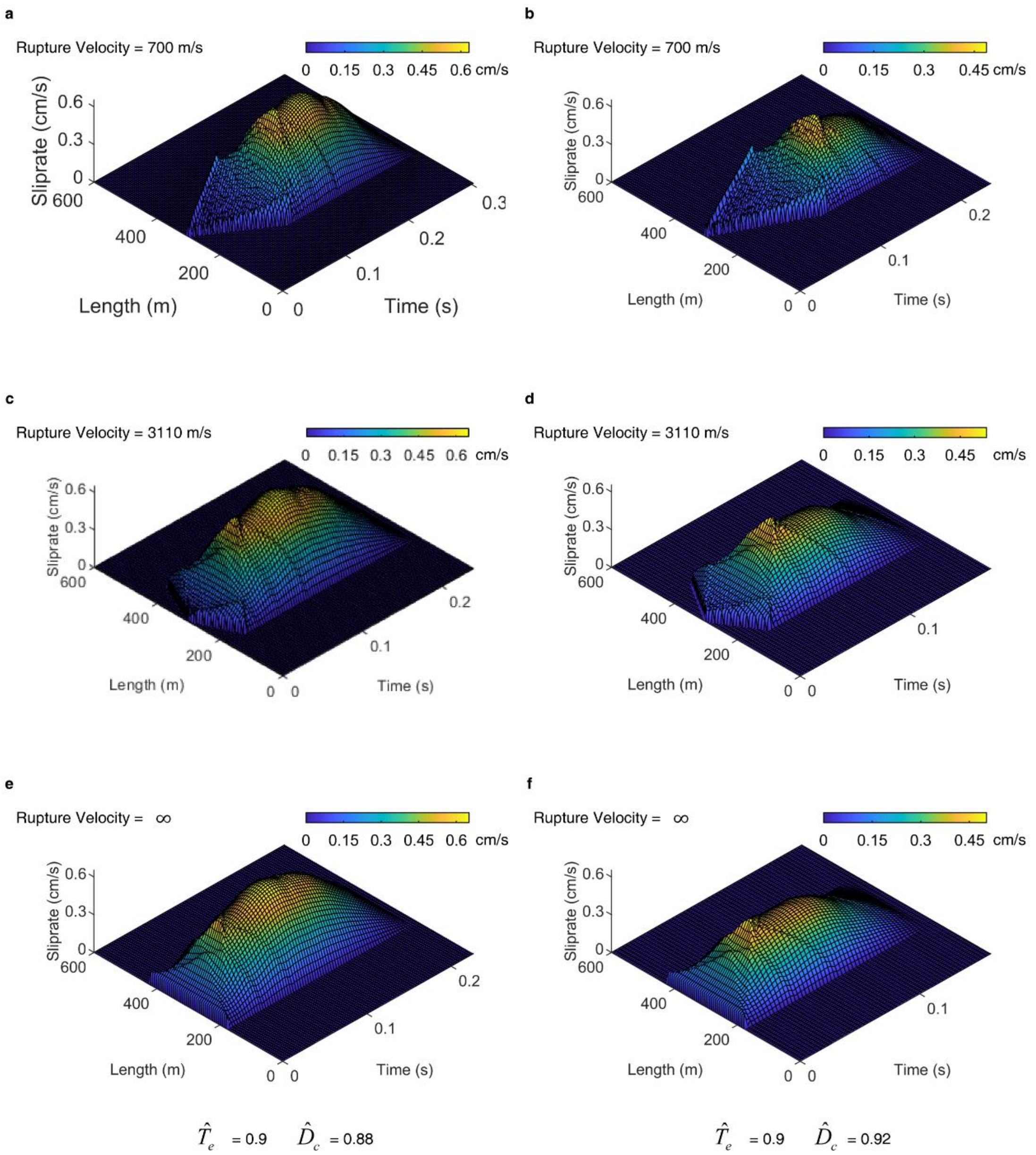

Slip rate on the fault as a function of space and time. **a**, SSAR with $\hat{D}_c$=0.88, $\hat{T}_e$=0.90, and rupture velocity 700 m/s. **b**, SSAR with $\hat{D}_c$=0.92, $\hat{T}_e$=0.90, and rupture velocity 700 m/s. **c**, SSAR with $\hat{D}_c$=0.88, $\hat{T}_e$=0.90, and rupture velocity 3110 m/s. **d**, SSAR with $\hat{D}_c$=0.92, $\hat{T}_e$=0.90, and rupture velocity 3110 m/s. **e**, SSAR with $\hat{D}_c$=0.88, $\hat{T}_e$=0.90, and rupture velocity ∞. **f**, SSAR with $\hat{D}_c$=0.92, $\hat{T}_e$=0.90, and rupture velocity ∞.

Fig. S13.

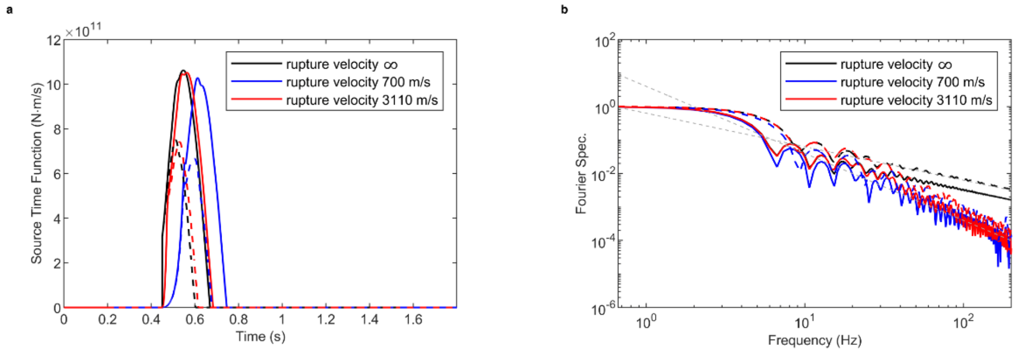

Source time function and the corresponding Fourier spectra of SSARs with different rupture velocities. a, Source time functions for SSARs with different rupture velocities. The black lines show the results of SSARs that rupture at the same time in the nucleation zone (with rupture velocity ∞). The blue lines show the results of a rupture velocity of 700 m/s. The red lines show the rupture velocity results at 3110 m/s. The solid lines show the results of SSARs with $\hat{D}_c=0.88$ and $\hat{T}_e=0.90$. The dashed lines show the results of SSARs with $\hat{D}_c=0.92$ and $\hat{T}_e=0.90$. **b**, The Fourier spectra for SSARs. The grey dashed line represents two lines with slopes of f^{-1} and f^{-2}.

Table S1.

	This work	M. G. Bostock (2015)[2]	A. M. Thomas (2016)[3]	S. R. Chestler (2016)[4]
Moment magnitude	0.7~2.2	1.0~2.61	~1	0.7~2.1
Stress drop (Pa)	2.6×10^{4}	1.53×10^{4}	$\sim10^{4}$	3×10^{4}
Rupture diameter (m)	240	600	200	275

Comparison between the estimated source parameters of Parkfield LFEs.

Table S2.

Model parameters	Sub-Rayleigh rupture	Self-arresting rupture	Slow self-arresting rupture
V_p (m/s)	6000	6000	6000
V_s (m/s)	3464	3464	3464
ρ (kg/m^3)	2670	2670	2670
T_u (MPa)	1.67~3.33	2.00~10.00	1.05~1.43
T_e (MPa)	1.0	1.0	1.0
T_i	$1.001\times T_u$	$1.001\times T_u$	$1.001\times T_u$
D_c (mm)	0.90~4.25	0.90~4.25	2.58~5.00
Q_s, Q_p	∞	∞	∞
Δs (m)	3.0	3.0	3.0
Rupture diameter (m)	180, 220, 260, 300	180, 220, 260, 300	180, 220, 260, 300
Domain size (grid cells)	200×200	200×200	200×200

Model parameters. V_p, P wave velocity; V_s, S wave velocity; ρ, density; T_u, breakdown stress drop; T_e, dynamic stress drop; T_i, initial stress in the nucleation zone; Q_s, S wave quality factor; Q_p, P wave quality factor; Δs, spatial dimensions of a grid cell in the computational model.

Table S3.

$\hat{D}_c$	Rupture velocity (m/s)	Duration (s)	Slip rate (m/s)	Moment Magnitude	Stress drop (Pa)
	700	0.3033	3.8734e-04	1.4687	3.5129e+04
0.88	3110	0.2406	4.9659e-04	1.4636	3.5729e+04
	∞	0.2268	5.2763e-04	1.4741	3.5764e+04
	700	0.2370	2.3650e-04	1.2545	1.6230e+04
0.92	3110	0.1737	3.3259e-04	1.2632	1.6716e+04
	∞	0.1599	3.6228e-04	1.2640	1.6728e+04

Source parameters of SSARs with different rupture velocities.

References

1 Uenishi, K. & Rice, J. R. Universal nucleation length for slip-weakening rupture instability under nonuniform fault loading. *J Geophys Res-Sol Ea* **108**, doi:Artn 204210.1029/2001jb001681 (2003).

2 Bostock, M. G., Thomas, A. M., Savard, G., Chuang, L. & Rubin, A. M. Magnitudes and moment-duration scaling of low-frequency earthquakes beneath southern Vancouver Island. *J Geophys Res-Sol Ea* **120**, 6329-6350 (2015).

3 Thomas, A. M., Beroza, G. C. & Shelly, D. R. Constraints on the source parameters of low-frequency earthquakes on the San Andreas Fault. *Geophys Res Lett* **43**, 1464-1471 (2016).

4 Chestler, S. R. & Creager, K. C. Evidence for a scale-limited low-frequency earthquake source process. *J Geophys Res-Sol Ea* **122**, 3099-3114 (2017).
