# Supplementary material for: The slow self-arresting nature of low-frequency earthquakes
Source: Nat Commun. 2021 Sep 15;12:5464. doi: 10.1038/s41467-021-25823-w (PMC8443596; doi:10.1038/s41467-021-25823-w)
Supplement: Supplementary file 3 — Description of Additional Supplementary Files [file 41467_2021_25823_MOESM3_ESM.pdf]

Description of Additional Supplementary Files

File name: Supplementary Data 1
Description: Simulated earthquakes source parameters. This file contains all the simulated earthquake source parameter results. The columns in the text file are the normalized $\hat{T}_e$; normalized $\hat{D}_c$; duration; slip; slip rate; seismic moment; stress drop; rupture style; and effective rupture diameter of each simulation earthquake. The rupture style is labelled as follows: 1 denotes a subRayleigh rupture; 2 is a self-arresting rupture; and 3 is a SSAR.
